# The first complete genome of *Fructilactobacillus vespulae*: strain Mu01, isolated from nectar of *Musa paradisiaca* L.

**DOI:** 10.1186/s12863-025-01329-y

**Published:** 2025-05-22

**Authors:** Manuel Zúñiga, Cristina Alcántara, Ángela Peirotén, Luis Andrés Ramón-Nuñez, Vicente Monedero, José María Landete

**Affiliations:** 1 Laboratorio de Bacterias Lácticas y Probióticos, Instituto de Agroquímica y Tecnología de Alimentos (IATA-CSIC), Av. Agustín Escardino 7, Paterna, 46980 Spain; 2 Departamento de Tecnología de Alimentos, National Institute for Agricultural and Food Research and Technology (INIA-CSIC), Carretera de La Coruña Km 7.5, Madrid, 28040 Spain; 3 Valencian Institute for Agricultural Research (IVIA), Valencia, 46113 Spain

**Keywords:** *Fructilactobacillus vespulae*, *Musa paradisiaca* L., *Lactobacillales*, Lactic acid bacteria, Nectar microbiota, Complete genome

## Abstract

**Objectives:**

*Lactobacillales*, commonly known as lactic acid bacteria (LAB), is an order of Gram-positive, facultatively anaerobic or microaerophilic bacteria characterized by their ability to ferment carbohydrates and produce lactic acid as a major metabolic byproduct. Many species within this group have significant roles in food fermentation, human health, and industrial applications. Here, we report the complete genome sequence of *Fructilactobacillus vespulae* Mu01, the first sequenced genome of this species. The complete genome sequence of *F. vespulae* Mu01 is expected to provide valuable insights into the genetics and metabolism of this little-characterized species.

**Data description:**

A novel strain of *Fructilactobacillus vespulae* was isolated from nectar of *Musa paradisiaca* L. during a survey for LAB associated with wild and cultivated plants in the metropolitan area of Valencia, Spain. A complete genome was obtained by sequencing with Nanopore long read technology. The genome consists of a chromosome of 1506092 bp and a plasmid of 42437 bp, presenting a GC content of 36 % and 31 %, respectively. The genome includes 1541 genes, with 1450 CDSs, 7 pseudogenes, 18 rRNA encoding genes, 63 tRNAs and 3 ncRNAs.

**Supplementary Information:**

The online version contains supplementary material available at 10.1186/s12863-025-01329-y.

## Objective

*Fructilactobacillus vespulae* is a Gram-positive, catalase-negative, facultatively anaerobic and heterofermentative bacterium belonging to the family *Lactobacillaceae*. *F. vespulae* was first named *Lactobacillus vespulae* [1]. The type strain, *F. vespulae* DCY75^T^ (= JCM 19742^T^), was isolated from the intestinal tract of a queen *Vespula vulgaris* wasp [1]. The species was later transferred to the genus *Fructolactibacillus* after a major revision of the classification of species in the families *Lactobacillaceae* and *Leuconostocaceae* [2]. Since its initial description, no further studies have been conducted on this species.

In a survey of lactic acid bacteria (LAB) associated with flowering plants in the metropolitan area of Valencia, Spain, diverse LAB species were isolated [3, 4]. Among them, strain Mu01 was classified as *F. vespulae* based on its 16 S rRNA sequence. Mu01 represents the second described isolate belonging to this species. Since no complete genome sequence had been determined for this organism, we decided to sequence the genome of *F. vespulae* Mu01.

## Data description

Strain Mu01 (CECT 31000 = DSM 117323 = CCM 9393) was isolated from aseptically collected nectar of *Musa paradisiaca* L. grown in an urban park in Valencia, Spain. Isolates were obtained as previously described [3, 4]. Preliminary identification of Mu01 was carried out by PCR amplification and partial sequencing of 16 S rRNA gene with primers 27 F (AGAGTTTGATCCTGGCTCAG) and 1492R (GGTTACCTTGTTACGACTT) [5]. The 16 S rRNA partial nucleotide sequence was compared with those available in the GenBank databases by using the BLAST tool (blastn) (https://blast.ncbi.nlm.nih.gov/Blast.cgi). Based on the BLAST result, Mu01 was identified as *F. vespulae* (99.9% identity with *F. vespulae* JCM19742^T^, Acc. Nº LC638721.1). Species of *Fructilactobacillus* are commonly isolated from insect intestines and flowers [2], a fact consistent with the origin of the Mu01 strain.

Sequencing libraries were prepared using Ligation Sequencing Kit V14 (SQK-LSK114, Oxford Nanopore) with barcoding SQK-NBD114.24. Libraries generated were sequenced on a MinION device using a FLO-MIN114 Vr10.4.1 flow cell (Oxford Nanopore) and duplex base-calling in super-accurate mode with Guppy (ver. 6.5.7). The mean read size was 3448.8 bp with a mean read quality of 22.8 as assessed with Nanoplot v1.36.2 [6]. A total of 51,414 reads were assembled using Flye v2.9.1 [7]. The assembly was deposited in DDBJ/ENA/GenBank (GCA_047324295.1; Data File 1 and 2) and annotated by the NCBI Prokaryotic Genome Annotation Pipeline (PGAP) [8]. In order to determine the taxonomic position of strain Mu01 more accurately, a core genome phylogeny was obtained (Data File 3). The genome sequences of 35 species of the most closely related genera, according to the phylogenetic reconstruction of family *Lactobacillaceae* were used [2]. Protein sequences were extracted, aligned and concatenated by using the desktop graphic application of VBCG v1.3 [9] (Data File 4). This program utilizes the corresponding homologous sequences of a preselected set of 20 core genes with the highest phylogenetic fidelity to build a concatenate alignment [9]. Best substitution model was selected by maximum likelihood in Mega 11.0.3. The maximum-likelihood tree was reconstructed with IQ-TREE using the LG + G4 + I + F + R substitution model [10]. Support values were obtained through the ultrafast bootstrap approximation implemented in IQ-TREE [11]. The analysis confirmed that *F. vespulae* is most closely related to *Fructilactobacillus sanfranciscensis*, consistent with previous findings based on 16 S rRNA gene phylogenetic reconstruction [1].

The completeness and contamination were assessed with CheckM [12] and reported as 98.45 and 0.16 %, respectively. Strain Mu01 harbors a 1,506,092 bp chromosome and a 42,437 bp plasmid (pFVMP1; data file 5). Analysis of Mu01 sequence identified 1450 protein encoding genes, 7 pseudogenes and, 84 RNA encoding genes, which included 6 rRNA operons, 54 tRNAs and 3 ncRNAs. These results align with data from the *Fructilactobacillus* genus, whose species have relatively small genomes, ranging from 1.23 Mbp to 1.44 Mbp [2]. Analysis with CRISPRCasFinder [13] revealed a type II-A CRISPR-Cas cluster consisting of an array of 48 repeats of 36 bp and a *cas* operon containing genes *cas9*, *cas1*, *cas2* and *csn2* (data file 6). Interestingly, the first spacer of the array targets a locus within gene R4B61_RS07660 located in the pFVMP1 plasmid. This may constitute a new example of self-targeting spacer [14]. In addition, the analysis with PHASTEST [15] identified a prophage related to other LAB bacteriophages (data file 7).

A search for antibiotic resistance genes using RGI v5.1.1 from CARD v3.3.0 [16] detected only a putative MFS efflux pump (R4B61_RS06345), whose homolog Bmr3 from *Bacillus subtilis* confers resistance to puromycin, tosufloxacin and norfloxacin [17]. Analysis of sugar utilization capabilities using API50 CH strips showed that Mu01 can utilize D-glucose, D-fructose, arbutin, esculin, gluconate and salicin. This limited range of fermentable carbohydrates is consistent with phenotypic data from insect- and nectar-associated lactobacilli. Like other members of heterofermentative lactobacilli and the *Fructilactobacillus* genus, Mu01 utilized fructose as an alternative electron acceptor, leading to the production of mannitol. In the Mu01 genome, a mannitol dehydrogenase gene (R4B61_RS00895) was identified adjacent to a putative fructose permease gene (R4B61_RS00900). Other phenotypic characteristics of the strain are listed in data file 8 (Table 1).

**Table 1:** Overview of data files/data sets

Label	Name of data file/data set	File types (file extension)	Data repository and identifier (DOI or accession number)
Data file 1	Complete chromosomal genome and annotation of *F. vespulae* Mu01	Genbank file (.gbk)	NCBI Reference Sequence (http://identifiers.org/refseq.gcf:GCF_047324295.1) [18]
Data file 2	Complete genome and annotation of *F. vespulae* Mu01 pFVMP1 plasmid	Genbank file (.gbk)	NCBI Reference Sequence (http://identifiers.org/refseq.gcf:GCF_047324295.1) [18]
Data file 3	Figure S1. Maximum likelihood phylogenetic tree of *F. vespulae* Mu01 and closely related species.	Portable Document Format file (.pdf)	DIGITAL.CSIC (https://doi.org/10.20350/digitalCSIC/17213) [19]
Data file 4	Concatenated alignment of 20 core protein sequences from *F. vespulae* Mu01 and closely related species used for the phylogenetic reconstruction	Fasta file (.fas)	DIGITAL.CSIC (10.20350/digitalCSIC/17213) [19]
Data file 5	Figure S2 and S3. Maps of chromosome and pFVMP1 plasmid of *F. vespulae* Mu01	Portable Document Format file (.pdf)	DIGITAL.CSIC (10.20350/digitalCSIC/17213) [19]
Data file 6	Table S1. Summary of Mu01 CRISPR-cas system.	MS Excel file (.xlsx)	DIGITAL.CSIC (10.20350/digitalCSIC/17213) [19]
Data file 7	Table S2. Summary of Mu01 prophage genetic characteristics.	MS Excel file (.xlsx)	DIGITAL.CSIC (10.20350/digitalCSIC/17213) [19]
Data file 8	Tables S3 and S4. Summary of phenotypic characteristics of Mu01	Portable Document Format file (.pdf)	DIGITAL.CSIC (10.20350/digitalCSIC/17213) [19]

## Limitations

This study provides the first complete genome sequence of a *F. vespulae* strain. The analyses are limited by the lack of complete genomic and metabolic data of other strains of this species. For this reason, it must be noted that the taxonomic assignment of strain Mu01 to *F. vespulae* may be modified in the future when the sequence of the genome of *F. vespulae* DCY75^T^ is determined. Furthermore, the availability of additional genomic sequences and the microbiological characterization of other strains of this species will provide insights into the evolution and ecological roles of this species.

## Supplementary Information

Supplementary Material 1.

Supplementary Material 2.

Supplementary Material 3.

Supplementary Material 4.

Supplementary Material 5.

## Data Availability

Complete chromosomal genome and annotation of F. vespulae Mu01 NCBI Sequence Read Archive (https://www.ncbi.nlm.nih.gov/nuccore/NZ_CP137138.1/) NCBI Sequence Read Archive (https://www.ncbi.nlm.nih.gov/nuccore/NZ_CP137139.1/)NCBI Reference Sequence (http://identifiers.org/refseq.gcf:GCF_047324295.1/) DIGITAL.CSIC (https://doi.org/10.20350/digitalCSIC/17213).

## Abbreviations

CARD: Comprehensive Antibiotic Resistance Database
LAB: Lactic acid bacteria
MFS: Major facilitator superfamily
RGI: Resistance Gene Identifier
