# Supplementary material for: The first complete genome of Fructilactobacillus vespulae: strain Mu01, isolated from nectar of Musa paradisiaca L
Source: BMC Genom Data. 2025 May 22;26:36. doi: 10.1186/s12863-025-01329-y (PMC12101011; doi:10.1186/s12863-025-01329-y)
Supplement: Supplementary file 1 — Supplementary Material 1. [file 12863_2025_1329_MOESM1_ESM.pdf]

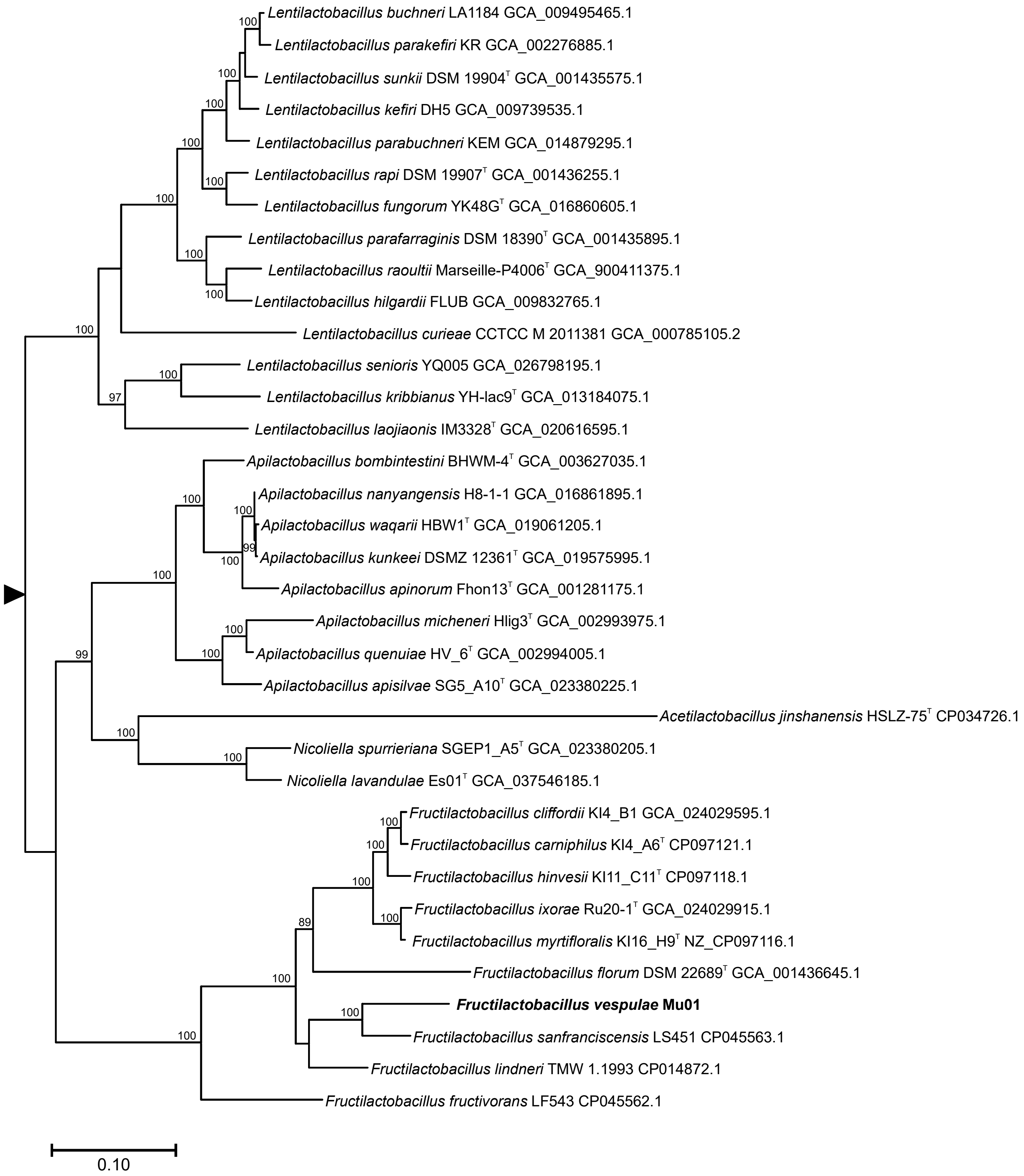


Figure S1. Maximum likelihood phylogenetic tree of core genes of species of *Fructilactobacillus* and closely related genera. Support values are given for those nodes with support higher than 75%. The tree has been arbitrarily rooted for ease of visualization.
