# Supplementary material for: The first complete genome of Fructilactobacillus vespulae: strain Mu01, isolated from nectar of Musa paradisiaca L
Source: BMC Genom Data. 2025 May 22;26:36. doi: 10.1186/s12863-025-01329-y (PMC12101011; doi:10.1186/s12863-025-01329-y)
Supplement: Supplementary file 5 — Supplementary Material 5. [file 12863_2025_1329_MOESM5_ESM.docx]

**Table S3.** Utilization of carbon sources by *F. vespulae* Mu01*

| **Carbon source** | **Growth** |
| --- | --- |
| Glycerol | - |
| Erythritol | - |
| D-Arabinose | - |
| L-Arabinose | - |
| D-Ribose | - |
| D-Xylose | - |
| L-Xylose | - |
| D-Adonitol | - |
| Methyl-ß-D-xylopyranoside | - |
| D-Galactose | - |
| D-Glucose | + |
| D-Fructose | + |
| D-Mannose | - |
| L-Sorbose | - |
| L-Rhamnose | - |
| Dulcitol | - |
| Inositol | - |
| D-Mannitol | - |
| D-Sorbitol | - |
| Methyl-α-D-mannopyranoside | - |
| Methyl-α-D-glucopyranoside | - |
| N-Acetylglucosamine | - |
| Amygdalin | - |
| Arbutin | + |
| Esculin/Ferric citrate | + |
| Salicin | + |
| D-Cellobiose | - |
| D-Maltose | - |
| D-Lactose | - |
| D-Melibiose | - |
| D-Saccharose | - |
| D-Trehalose | - |
| Inulin | - |
| D-Melezitose | - |
| D-Raffinose | - |
| Starch | - |
| Glycogen | - |
| Xylitol | - |
| Gentiobiose | - |
| D-Turanose | - |
| D-Lyxose | - |
| D-Tagatose | - |
| D-Fucose | - |
| L-Fucose | - |
| D-Arabitol | - |
| L-Arabitol | - |
| Potassium gluconate | + |
| Potassium 2-ketogluconate | - |
| Potassium 5-ketogluconate | - |

* Cells were grown in MRS agar plates at 30°C and resuspended in phosphate buffer saline to inoculate API 50 CHL medium (Biomerieux), which was dispensed in API 50 CH strips (Biomerieux) as recommended by the manufacturer. Strips were incubated at 30 °C for 48 h.

**Table S4.** Growth characteristics of *F. vespulae* Mu01

|  | Growth range | Optimal | Isomer (g/L) |
| --- | --- | --- | --- |
| Temperature* | 15-37°C | 30°C |  |
| pH* | 3.5-9 | 6-7 |  |
| NaCl* | 0-7% | 0% |  |
| Lactic acid production** |  |  | D-lactic 0.81±0.30  L-lactic 8.19±1.51 |

* Growth in MRS at the different conditions tested was checked daily for five days.

** Production of D and L lactic was measured in MRS-fructose supernatant using ENZYTEC^TM^ D-/L-Lactic acid enzymatic kit according to manufacturer instructions (ref#E1255, R-Biopharm AG, Darmstadt, Germany). Mu01 was subcultured twice in MRS (24h, 30°C), the supernatant was filtered (0.22μm) and diluted 1:50 and 1:10 to be used as sample. Determinations were performed in duplicate.
