# Supplementary material for: The first complete genome of Fructilactobacillus vespulae: strain Mu01, isolated from nectar of Musa paradisiaca L
Source: BMC Genom Data. 2025 May 22;26:36. doi: 10.1186/s12863-025-01329-y (PMC12101011; doi:10.1186/s12863-025-01329-y)
Supplement: Supplementary file 2 — Supplementary Material 2. [file 12863_2025_1329_MOESM2_ESM.pdf]

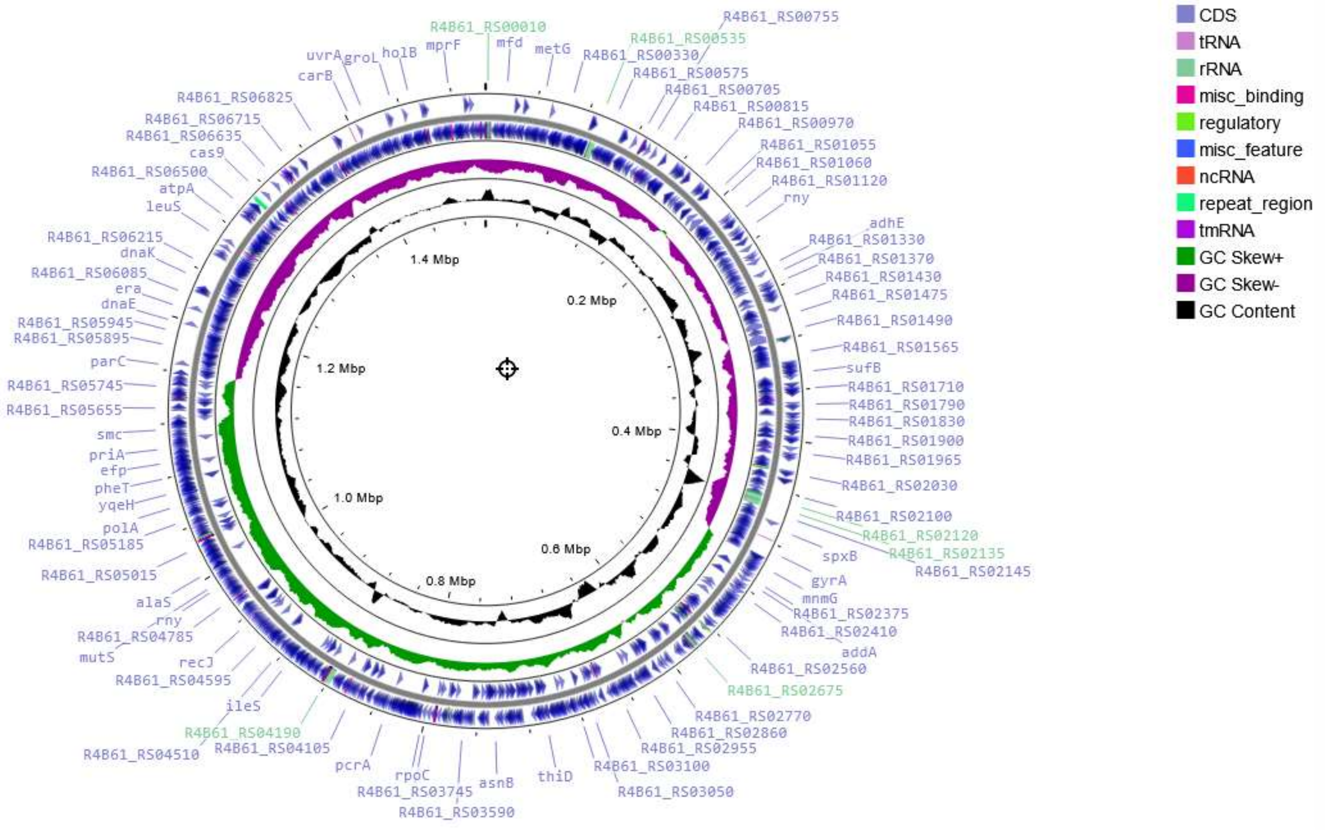

Figure S2. *Fructilactobacillus vespulae* Mu01 chromosome. The map was generated with Proksee (Grant et al. Proksee: in-depth characterization and visualization of bacterial genomes. Nucleic Acids Research, 2023, gkad326, https://doi.org/10.1093/nar/gkad326

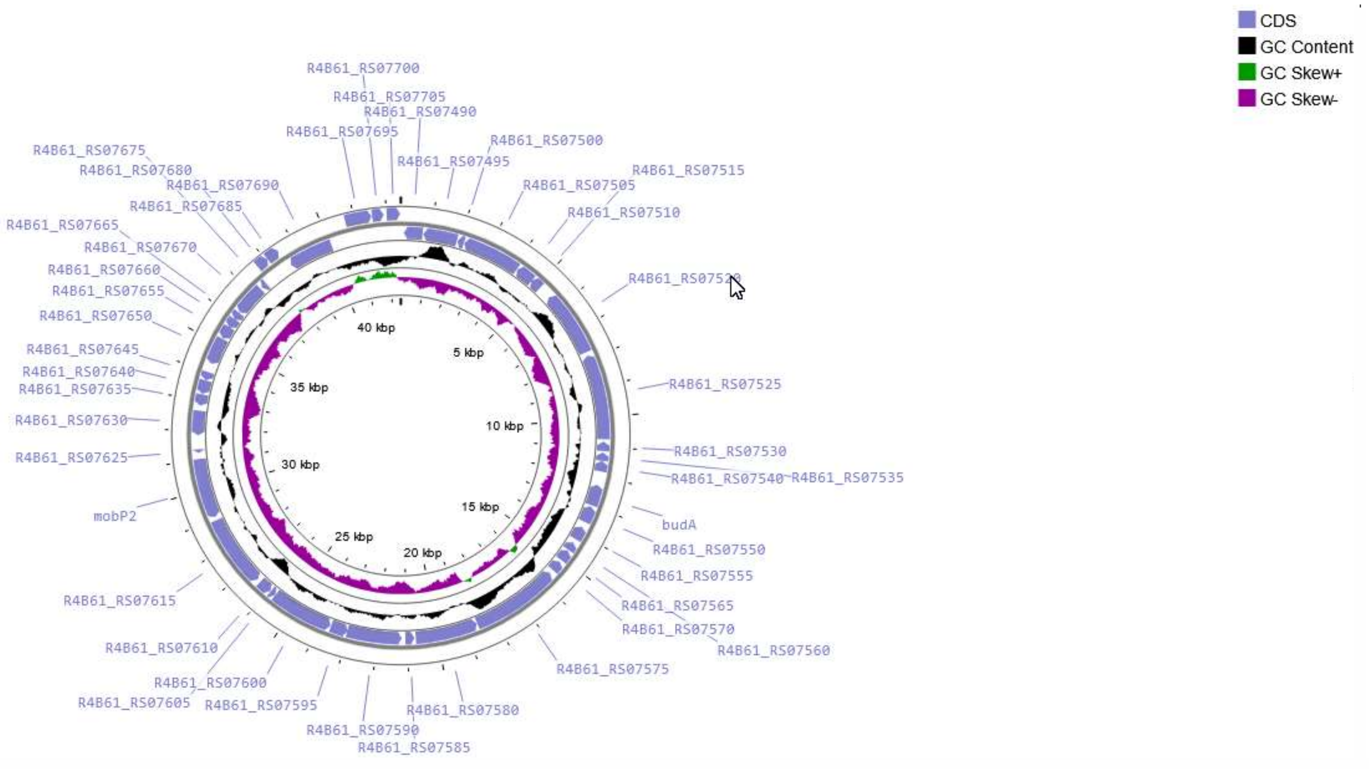

Figure S3. *Fructilactobacillus vespulae* Mu01 plasmid pFVMP1. The map was generated with Proksee (Grant et al. Proksee: in-depth characterization and visualization of bacterial genomes. Nucleic Acids Research, 2023, gkad326, https://doi.org/10.1093/nar/gkad326
